# Defence response mobilization in response to provocation or imagery of interoceptive sensations in adolescents with chronic pain: a study protocol

**DOI:** 10.1097/PR9.0000000000000680

**Published:** 2018-09-11

**Authors:** Piotr Gruszka, Luca Schaan, Dirk Adolph, Christiane A. Pané-Farré, Christoph Benke, Silvia Schneider, Tanja Hechler

**Affiliations:** a Department of Clinical Child and Adolescent Psychology, Ruhr-Universität Bochum, Bochum, Germany; b Department of Clinical Child and Adolescent Psychology and Psychotherapy, University of Trier, Trier, Germany; c Department of Biological and Clinical Psychology, University of Greifswald, Greifswald, Germany

**Keywords:** Pediatric, Chronic pain, Abdominal pain, Headache, Conditioning, Anticipation, Provocation, Imagery

## Abstract

**Introduction:**

Fear of pain seems to be a key factor in the development and maintenance of chronic pain and pain-related disability. Interoceptive fear conditioning is assumed to constitute an important mechanism in the origins and maintenance of fear of pain. If conditioned stimuli such as internal bodily sensations are repeatedly paired with pain (unconditioned stimulus), they in turn elicit a conditioned fear response, including defence mobilization such as startle modulation and changes in heart rate and electrodermal activity. Research into emotional imagery suggests that defensive responses can also be elicited through imagery of fear scripts.

**Objectives:**

We present 2 novel paradigms adapted from research on anxiety disorders, which allow to test, if perceived or imagined sensations locally proximal to the main pain location trigger heightened defence response mobilization in adolescents with chronic headaches and abdominal pain.

**Methods:**

The *provocation* paradigm includes the anticipation and provocation of locally proximal and locally distal interoceptive sensations through disorder-specific muscle tensing tasks (tightening the neck or the abdominal muscles). The *imagery* paradigm includes 3 imagery scripts (standard neutral, standard fear, and disorder-specific). Startle probes are presented in both paradigms. Defence response mobilization is assessed using psychophysiological measures (startle response modulation, skin conductance level, and heart rate), as well as self-reported measures of fear.

**Perspective:**

The paradigms will give insight into the defence response of adolescents with chronic pain, when confronted with or imagining interoceptive sensations. Results may inform the improvement of clinical interventions aimed to decrease fear of bodily sensations such as interoceptive exposure or interoceptive imagery exposure.

## 1. Introduction

Ample evidence suggests that pain-related fear is a core maintaining factor of the experience of ongoing pain and a risk factor for the development of pain in adults.^31,45,46^ There is also some evidence in adolescents for this association.^3,10,42,43^ Pain as a survival-relevant threat has been demonstrated to activate defensive survival networks in the brain that initiate and orchestrate physiological and behavioural defensive responses to protect the individual from potential harm (eg, a potential tissue damage). Evidence from experimental studies suggests that defensive responses (eg, autonomic arousal and motor response preparation) may be elicited by early interoceptive (ie, internal bodily) cues.^36^ Thus, from a fear learning perspective, interoceptive sensations may become predictors of pain and elicit defensive responses subsequently. This form of learning, in which an interoceptive sensation (such as stomach grumbling) becomes a conditioned stimulus (CS) when repeatedly paired with an unconditioned stimulus (US; such as pain), is referred to as interoceptive fear conditioning^15^ and plays a central role in current learning theory accounts of panic disorder.^6^ Interoceptive fear conditioning may be, in particular, relevant for the acquisition of defensive responses to internal bodily sensations in individuals with chronic pain.^15^ There is some evidence that particularly interoceptive sensations within the same physiological system or at the same anatomical location as the pain (US) can function as a CS. In a recent study in healthy adults, nonpainful visceral sensations (lowest intensities of innocuous esophageal balloon distension) paired with electrical painful stimuli elicited a conditioned threat response as indicated by a potentiated startle reflex that indexes defensive motor response preparation.^48^ Similarly, in another experiment, Ceunen et al.^11^ demonstrated that nonpainful esophageal stimuli can evoke a conditioned threat response in healthy adults, when associated with painful esophageal stimuli at the same anatomical location. Bradley et al.^8^ demonstrated that healthy participants respond with a defensive response (potentiated startle), when threatened by the possibility of receiving a mild electric shock. The assumption that interoceptive sensations proximal to the main pain region trigger fear responses in individuals with chronic pain has been rarely tested experimentally, however. Flack et al.^19^ showed in a recent quasi-experimental study that adolescents with chronic abdominal pain (CAP) (N = 40, aged 11–18 years) self-reported increased fear and avoidance when confronted with locally proximal sensations through muscle tensing tasks (tighten the stomach and tighten the corrugator supercilii muscle). Whether these self-reports also converge with psychophysiological defence responses is yet unknown.

Not only perceived interoceptive sensations but also mental imagery of interoceptive sensations has been shown to elicit defence response mobilization.^35^ “Mental imagery occurs when perceptual information is accessed from memory, giving rise to the experience of ‘seeing with the mind's eye’…,”^27^ thus mental imagery can be defined as an experience of perception in the absence of a concurrent sensory input.^28^ It has been proposed that emotional images consist of associative networks that include sensory, semantic, and response information (eg, behavioural, autonomic, and reflexive responses).^29^ As such, mental imagery of interoceptive sensations may lead to the activation of response information and thus elicit measurable autonomic and reflexive responses. In line with that, it has been demonstrated in adults that mental imagery of fear-relevant interoceptive sensations (CS), but not neutral imagery scripts, result in increased subjective fear and tidal volume when associated with inhalation of CO_2_ (US) in healthy subjects.^14,44^ Moreover, a recent study demonstrated that idiographic disorder-specific aversive imagery of panic attacks elicited greater startle potentiation in adult patients with panic disorder than in healthy adult participants.^35^ However, it has not yet been investigated whether the imagination of interoceptive sensations proximal to the main pain region also elicit a mobilization of defence responses in adolescents with chronic pain. Thus, there is only scarce evidence showing that the anticipation, provocation, or imagery of interoceptive sensations at the pain site can trigger a defence response mobilization.

### 1.1. Aim of the article

To close this research gap, we present here a study protocol describing 2 paradigms adapted from research on anxiety disorders^13,36^ to evaluate defence response mobilization in response to the anticipation, provocation, and imagery of interoceptive sensations in adolescents with chronic headaches (CH) and in adolescents with abdominal pain.

### 1.2. Study hypotheses

#### 1.2.1. Provocation paradigm

We hypothesize that adolescents with chronic pain will exhibit increased defence response mobilization when anticipating and perceiving locally proximal sensations to the main pain site (pain-specific tasks) compared with locally distal interoceptive sensations. We expect that these participants will also report higher self-report ratings of avoidance, escape, and fear. Participants with chronic pain should also exhibit a stronger defence response mobilization than healthy controls (HCs) during these pain-specific tasks.

#### 1.2.2. Imagery paradigm

We hypothesize that adolescents with chronic pain will display increased defence response mobilization during the imagery of sensations typically associated with the main pain (pain-specific aversive imagery) compared with neutral imagery. Pain-specific aversive imagery will also result in higher self-reported ratings of fear of pain in these participants. We expect aversive fear imagery scripts to elicit a stronger defence response than neutral imagery scripts in participants with chronic pain and in HCs.

### 1.3. Exploratory research questions

We will also investigate predictors of the magnitude of defence response mobilization such as the level of pain during the tasks, severity of chronic pain, and anxiety sensitivity. Comparing the defence response mobilization during the imagery of pain-specific aversive scripts with responses during the imagery of fear scripts might enable specifying defence response mobilization in relation to the threat imminence continuum.^16^

### 1.4. Relevance of the paradigms

The 2 paradigms enable to show if adolescents with chronic pain display pain-related defence response mobilization when confronted with or imagining interoceptive sensations proximal to the main pain location. These findings may have high clinical relevance, as they may suggest that innocuous interoceptive sensations (experienced or imagined) have the capacity to trigger fear responses and subsequent avoidant or recuperative behaviour in adolescents with chronic pain. A recent study in N = 126 adolescents with chronic pain suggests that interoceptive exposure may be particularly effective for adolescents with initial high fear of pain before treatment.^20^ The conception of interoceptive exposure tasks is, however, yet understudied. The symptom provocation tasks should be able to elicit substantial fear responses.^22^ Our study is the first to address this research question and may thus provide the basis for developing specific tasks to be included into interoceptive exposure, such as symptom provocation or imagery-based tasks.

## 2. Methods

### 2.1. Study design

The study design consists of 2 quasi-experiments (*provocation* and *imagery*) investigating the effects of anticipation, provocation, and imagination of interoceptive sensations on psychophysiological and self-reported measures in 3 groups of adolescents (11–18 years). Adolescents with CH, adolescents with CAP, and HCs are compared in their defence response mobilization during anticipation, provocation, and imagery of interoceptive sensations (between- and within-subject design). Presentation order of the paradigms (*provocation* vs *imagery*) is counterbalanced for each group with a break of 10 minutes between each paradigm.

The *provocation* paradigm consists of 3 muscle tensing tasks to induce interoceptive sensations: (1) the *tighten stomach task* (S), (2) the *neck task* (N), and (3) the *safe/neutral* control *task*, ie, *clenching the fist* (F) (described in more detail below). The S condition should be particularly aversive for participants with CAP and less aversive for adolescents with CH and for healthy adolescents. The N condition should be aversive for participants with CH but less aversive for participants with abdominal pain and healthy adolescents. The s*afe task—clenching the fist (F)* condition is designed to be nonaversive for all groups (safe control task).

The *imagery* paradigm consists of 3 conditions: *pain-specific imagery* (P), *fear imagery* (F), and *neutral imagery* (N) (described in more detail below). *Pain-specific imagery* is expected to be aversive for adolescents with chronic pain, *fear imagery* should be aversive for all groups, and *neutral imagery* is designed to be nonaversive for all groups.

### 2.2. Inclusion/exclusion criteria

Inclusion criteria are as follows: Adolescents included if they are aged 11 to 18 years and are German speaking.

We defined 3 study groups a priori as well as 2 additional mixed groups during the recruitment of participants because of high comorbidity between abdominal pain and headaches^18^: (1) adolescents with CH (headaches at least once per week for at least 3 months; not suffering from abdominal pain more than 2 times per month; and no impairment due to abdominal pain), (2) adolescents with CAP (abdominal pain at least once per week for at least 3 months; not suffering from headaches; and no impairment due to headaches), (3) a HC group (do not suffer from abdominal pain or headaches more than 2 times per month, nor are they impaired by pain or do have a history of chronic pain treatment), (4) adolescents with CH as the primary pain and additional abdominal pain (abdominal pain may be less pronounced while criteria for the headache group need to be fulfilled), and (5) adolescents with CAP as the primary pain and additional headaches (headaches may be less pronounced while criteria for the abdominal pain group need to be fulfilled). The 2 additional mixed groups were not included in the sample size calculation and are therefore only exploratory. Once, the data are completed, supplementary analyses on the effect of the co-occurrence of both types of pain on defensive responses can be conducted.

Adolescents and their parents must give written informed consent to take part in the study, unless the adolescent is 18 years of age. Exclusion criteria comprise a diagnosis of posttraumatic stress disorder (assessed via the posttraumatic stress disorder section of the Mini-Dips for DSM-5,^33^ which was adapted for adolescents) or organic causes of pain (assessed via an interview). Adolescence with chronic pain and comorbid anxiety disorders are not excluded, as this method addresses a research question on the verge between chronic pain and anxiety disorders, ie, fearful responses to internal bodily sensations.^5,38^

### 2.3. Ethical approval

A study using the here proposed paradigms was approved by the ethics committee of the University of Bochum (no. 151/152) and Trier. Participants receive financial compensation of 20 € in Bochum (in addition to having the opportunity of taking part in a treatment program) or 40 € in Trier (without the opportunity of taking part in a treatment program) for study participation.

### 2.4. Measures

#### 2.4.1. Defence response mobilization

The defence response mobilization is assessed in both paradigms using psychophysiological measures (startle response magnitude, skin conductance level (SCL), and heart rate [HR] [variability]), as well as self-reported measures of fear. All psychophysiological measures in the ongoing study are measured using a BrainAmp ExG amplifier with a sampling rate of 1000 Hz.

##### 2.4.1.1. Primary measure

###### 2.4.1.1.1. Startle response magnitude

The primary measure is the startle eyeblink response, measured by assessing muscle electrical activity (EMG) of the orbicularis oculi beneath the left eye during the reflex blink using two 13/5-mm Ag/AgCl electrodes with Signacreme electrode cream (Parker Laboratories, Inc, Fairfield, NJ). Relevant skin areas are previously treated with a slightly abrasive gel (Nuprep; Weaver and Company, Aurora, CO) and cleaned with distilled water. The startle reflex is elicited by binaurally delivered bursts of white noise with an intensity of 95 dB (A-weighted) and a duration of 50 ms (rise/fall time <1 ms).^4^ The decision to use bursts of white noise with an intensity of 95 dB was made based on previous studies with adolescents.^23^ EMG startle data are prepared for data analysis according to Ref. 41.

##### 2.4.1.2. Secondary psychophysiological measures

###### 2.4.1.2.1. Skin conductance

Skin conductance is recorded continuously with two 13/5-mm Ag/AgCl electrodes with skin conductance electrode paste (TD-246; MedCat, Tucson, AZ) placed on the medial phalanxes of the index and middle fingers of the nondominant hand. A direct constant voltage of 0.5 Vdc is used.

###### 2.4.1.2.2. Heart rate

Electrocardiogram is recorded continuously according to the Einthoven lead II setup using disposable self-adhesive solid gel Ag/AgCl electrodes (40-mm diameter). Relevant skin areas are previously treated with an abrasive gel (Nuprep; Weaver and Company) and cleaned with distilled water. R-Spikes can be detected using available software solutions (eg, Anslab).

###### 2.4.1.2.3. EMG

Surface EMG signals are introduced as a manipulation check to test whether adolescents follow instructions to tense the respective muscle group in the provocation paradigm. Tensing the neck muscles is controlled for by recording surface EMG at the superior fibers of the trapezius muscle (measured lateral to the second cervical vertebrae) using disposable self-adhesive solid gel Ag/AgCl electrodes (40-mm diameter). Tightening the abdominal muscles is controlled for by recording EMG at the left lower rectus abdominis (2 cm lateral and caudal to the umbilicus, and left obliquus externus; over the tip of the eighth rib and angled diagonally in the direction of the muscle fibers^37^). Clenching of the fist is controlled for by recording surface EMG signals in the middle of the musculus brachioradialis of adolescents' dominant arm (measured 5 cm distal to the elbow).

#### 2.4.2. Self-reported secondary measures

##### 2.4.2.1. Provocation

###### 2.4.2.1.1. Self-reported fear

Adolescents report their fear after each provocation trial by drawing a fear curve (*x*-axis = time, *y*-axis = fear: 0 [“No fear at all”] to 10 [“Strongest fear imaginable”]) representing changes in fear during 3 phases of the provocation paradigm (anticipation, exposure, and recovery) on an Intuos PEN & Touch S Tablet (Wacom).

###### 2.4.2.1.2. Self-reported avoidance tendency

The urge to avoid the task and to leave the situation is assessed with 2 questions on a visual rating scale ranging from 0 (“Not at all”) to 100 (“Extremely”) after each provocation trial.

###### 2.4.2.1.3. Similarity of interoceptive sensations

The similarity between interoceptive sensations during the tasks and sensations which participants feel before the pain onset in their everyday lives is assessed on a visual rating scale ranging from 0 (“Not similar at all”) to 100 (“Very similar”) with the question “How similar were your physical sensations during the exercise to those that you usually feel before the pain?”. To test the validity of the paradigm, participants are also asked to mark body locations, where they have felt sensations during the provocation task on a schematic drawing showing a human torso and head (inspired by the method of pain drawings).^32^

###### 2.4.2.1.4. Self-reported pain

Pain is rated on a numeric rating scale from 0 (“No pain at all”) to 10 (“Strongest pain imaginable”) retrospectively at the end of the provocation trials.

##### 2.4.2.2. Valence and arousal of the imagery scripts

*Valence* and *arousal* are assessed using the Self-Assessment Manikin.^7^ *Vividness of imagery*, *fear*, *fear of pain*, and the *urge to leave* the *situation* are assessed on a visual rating scale from 0 (“Not at all”) to 100 (“Extremely”) after trial presentation retrospectively. Pain is rated on a numeric rating scale from 0 (“No pain at all”) to 10 (“Strongest pain imaginable”) retrospectively after trial presentation.

### 2.5. Materials

The paradigms were programmed in Python 2.7 using libraries included in PsychoPy 1.83.04.^39^

#### 2.5.1. Equipment

Basic psychophysiological laboratory equipment is necessary to conduct the experiments. A detailed description of a basic psychophysiological setup can be found elsewhere.^12^

#### 2.5.2. Test chamber

Equipment in the test chamber include a human psychophysiological acquisition system with a minimum of 6 acquisition channels and a minimum sampling rate of 1000 Hz to assure unbiased assessment of the startle reflex (eg, BRAINAMP ExG), consumable supplies, a monitor for stimulus presentation, high-quality headphones, and a comfortable armchair so that the experiment is tolerable for adolescents and a video camera/webcam for subject monitoring.

#### 2.5.3. Control room

Equipment in the control room include a personal computer with a low latency sound card running Windows or Linux, which is connected to the monitor in the test chamber and a second monitor in the control room. As an alternative to a low latency sound card, a white noise sound generator can be used for eliciting startle responses. A parallel port is used for inserting triggers during the presentation of startle probes.

### 2.6. Procedure

**Figure 1.**
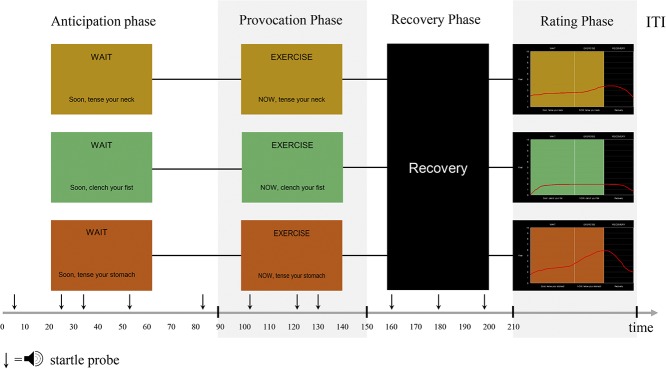
The 4 phases of a provocation trial. ITI, intertrial interval; time, time in seconds.

**Figure 2.**
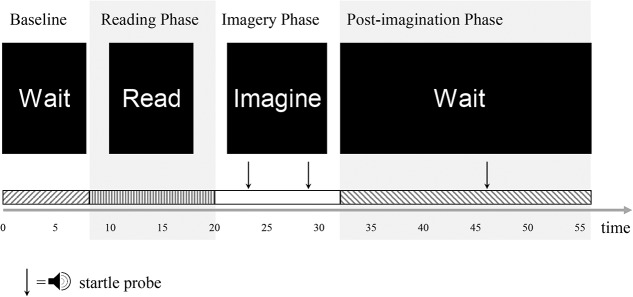
The 4 phases of an imagery trial. time, time in seconds.

Study participants are informed about the study and screened for eligibility on the phone. Participants have the possibility to ask questions about the study at that time. If they are eligible and interested to take part in the study, they receive additional study information and the consent form through email and are asked to fill out several questionnaires (eg, sociodemographic variables, pain, and anxiety-specific questionnaires) before coming to the laboratory. Once participants arrive in the laboratory, written consent is provided by the adolescent and his/her parent. Participants are asked to sit down in a comfortable reclining chair. Electrodes are attached after filling out a sensation questionnaire (see below for details). Electric signals are tested, and the experiment is started. After the habituation phase of 1.5 minutes with 5 startle probes (to habituate to a stable startle baseline), participants start with either the *provocation* or the *imagery* paradigm. Procedures for the *imagery* and *provocation* paradigms are described in more detail below (Figs. 1 and 2). A 5-minute resting phase follows both paradigms.

#### 2.6.1. Provocation

Three muscle tensing tasks are used during the experiment: *Tighten stomach task (S)*, *tension the neck task (N)*, and s*afe comparison task*: *clenching the fist (F).*

In the S condition, participants are instructed to tense their abdominal muscles for 60 seconds (respective muscle: musculus rectus abdominis). In the N condition, participants are instructed to tense their neck for 60 seconds (respective muscle: musculus trapezius). If participants have difficulties to tense their neck muscles, they are instructed to press their head against the backrest of their chair. Care is taken to ensure that the adolescents could continue to look on the screen, and electrodes do not receive any pressure. In the F condition, participants are asked to clench their fist of their dominant hand for 60 seconds (respective muscle: musculus brachioradialis).

Each trial consists of 4 phases: anticipation, exposure, recovery, and rating (Fig. 1). In the anticipation phase (90 seconds), a task-specific screen background colour (eg, green) is presented with instructions that the stomach/neck/fist tensing task will start shortly. In the exposure phase (60 seconds), participants are shown the same screen colour with instructions to tense the respective muscle groups. The recovery phase is indicated by a black screen with white text indicating a “Recovery” phase (60 seconds). After these 3 phases, participants are asked to draw a curve representing their fear during the 3 phases and rate their urge to avoid and leave the situation. Each trial ends with an intertrial interval of 3 to 9 seconds (Fig. 1).

The anticipation phase is accompanied by 5 startle probes. The first startle probe is delivered 5 or 15 seconds after the anticipation onset. The interval between 2 startle probes lasts for 10, 20, or 30 seconds (counterbalanced). The exposure phase is accompanied by 3 startle probes. The first startle probe is delivered at 5, 15, or 25 seconds after the exposure onset, and the interval between 2 startle probes lasts for 10, 20, or 30 seconds (counterbalanced). The recovery phase is also accompanied by 3 startle probes. The first startle probe is delivered 5, 15, or 25 seconds after the recovery onset, and the interval between 2 startle probes lasts for 10, 20, or 30 seconds (counterbalanced).

The trials are presented in one of the following different orders: (1) S F N F N S N S F, (2) N S F S F N F N S, and (3) F N S N S F S F N. The provocation paradigm lasts approximately 35 minutes (Fig. 2). Participants are instructed to perform the 3 muscle tensing tasks before the experiment (eg, “please tense your abdominal muscles now, so that we can check the signal”) and to practice the exercises while breathing naturally. The experiment is started only after the study investigator confirms appropriate EMG signals during these tasks.

#### 2.6.2. Imagery

Three different scripts with a length of approximately 20 words are used in each imagery condition: *pain-specific imagery* (P), *fear imagery* (F), and *neutral imagery* (N). Two of the *aversive fear imagery* scripts are based on previously published scripts.^13^ Scripts in the “*pain-specific imagery*” condition are individualized for each participant using a newly developed sensation questionnaire. Participants are asked about the frequency of different sensations in the pain area preceding pain episodes (or pain increases) and rate the unpleasantness and mark the location of the 3 most unpleasant sensations thereafter. “Standard” pain-specific aversive scripts (eg, “I feel a slight tension in the middle of my stomach. I can also feel a prickling in the middle of my stomach.”) are adapted by substituting the body sensation and location in the second sentence in each script with one of the individualized (unpleasant) sensations and associated locations. Thus, in the aforementioned “standardized” script, “prickling sensation” and “middle of my stomach” might be substituted with “tension” and “upper abdomen.”

After receiving detailed instructions, participants rate the emotional valence and arousal levels of each script using a computerized version of the Self-Assessment Manikin.^7^ Endpoints of valence and arousal ratings are specified according to previously published studies in children.^34^ Thereafter, instructions are presented to participants, including situations of being absorbed in everyday situations. Participants are instructed to imagine the situations/sensations described in the scripts vividly (as if they would really engage in them). They are instructed to imagine the situations with open eyes. The *imagery* trials begin with a test trial in which an additional script is presented. Each script is presented twice in a counterbalanced fashion, totalling 19 trials. All trials consist of a baseline (6, 7, or 8 seconds), a reading (12 seconds), an imagery (12 seconds), and a postimagery phase (23, 24, or 25 seconds). In 50% of the trials, startle probes are delivered at 2 time points during the imagery phase, one at second 3, 4, or 5 and another one at second 9, 10, or 11. In approx. 25% (4 of 18) of trials, one startle probe is delivered at second 3, 4, or 5, and in another approx. 25% (5 of 18) of trials, one startle probe is delivered at second 9, 10, or 11 of the imagery phase. One startle probe is delivered in 50% of the postimagery phases at second 13, 14, or 15 (Fig. 2). Scripts are presented at the end of the trials once again, and participants rate the scripts on 5 domains (vividness of imagery, fear, fear of pain, desire to avoid the situation, and pain). The imagery paradigm lasts approximately 22 minutes.

### 2.7. Feasibility

#### 2.7.1. Provocation paradigm

A previous pilot study in adolescents with CH or CAP has already tested whether the provocation of internal bodily sensations results in increases in self-reported fear.^19^ Flack et al. showed that the perception of proximal interoceptive sensations appears to activate the fear system (measured by self-report) in adolescence with CAP. Adolescents with CH did not report higher fear or avoidance ratings. According to Flack et al., this effect may possibly be explained by the choice of the frown task. The corrugator supercilii muscle is used in everyday communication processes and is thus also activated during different emotional states. Frowning may have not elicited locally proximal interoceptive sensations in adolescents with chronic pain.

Based on these pilot results, various adjustments were made. The frowning task (contracting the corrugator supercilii muscle) was replaced by the “tensing the neck” task. In addition, self-report ratings were extended with psychophysiology measures (EMG, electrodermal activity, and ECG)—in particular, the assessment of fear-induced startle potentiation as an indicator of defence response mobilization. In addition, the following minor adjustments were made: The length of each phase was optimized (extension of the anticipation phase from 3 to 90 seconds and reduction of the provocation phase from 180 to 60 seconds). To ensure the validity of the paradigm, we added an additional question (“How similar were your physical sensations during the exercise to those that you usually feel before the pain?”) and an additional task (marking body locations, where participants have felt sensations during the provocation phase). Also, self-report measures were extended (eg, fear curve).

#### 2.7.2. Imagery paradigm

The sensation questionnaire, imagery scripts, and recruitment strategies were tested in a pilot study, including 14 adolescents (age: mean [M] = 13.8, SD = 2.4) with CH or CAP recruited from the community and 20 HCs aged 11 to 18 years (age: mean [M] = 14.2, SD = 2.24). Pain was considered chronic if it was experienced at least once per week during the past 3 months. Results of our pilot study showed markedly higher self-reported avoidance ratings in adolescents with CAP (N = 8) and adolescents with CH (N = 6) compared to HCs (N = 20) during the imagery of interoceptive sensations at the pain location.

Several aspects of the imagery paradigm were adapted: We replaced one neutral imagery script, which showed differing valence ratings. Instructions are now read aloud by the study investigator (instead of including recorded audios in the experiment) to make sure that all participants feel comfortable to ensure high-quality psychophysiological data. One participant reported imagining the scripts during the reading phase and ruminating about her ratings during the imagery phase. Therefore, participants are instructed explicitly not to imagine the situation while reading the scripts but only during the imagery phase. We have also moved self-report ratings to the end of the trial to prevent this kind of behaviour. Many participants had difficulties to list 6 interoceptive sensations preceding pain episodes. Therefore, only the second sentence of each script is individualized to ensure that study participants have to list only 3 sensations preceding pain episodes.

##### 2.7.2.1. A priori calculated sample size

Our analysis is based on our primary hypothesis that interoceptive stimuli proximal to the main pain will elicit greater defence response mobilization than distal stimuli in adolescents with chronic pain compared to healthy children using startle magnitude as the primary outcome. Previous studies in adults comparing startle magnitude during interoceptive threat between healthy adults high and low in anxiety sensitivity found small to medium effect sizes (eg, Ref. 36 [eta-squared = 0.106]). The stability of the emotion-modulated startle response has been shown to be high with correlations between measurements of *r* = 0.5.^30^ We based our sample size calculation on study results from adults. Given that increased startle overall magnitude has previously been reported during adolescence,^40^ this results in a conservative estimate of the requested sample size. At an alpha level of 0.05, the sample size of 33 per group (total sample size = 99) is suitable to detect the within–between interaction effect (moderate size, f = 0.145) with a power of 0.80.^17^ Taking a dropout of approx. 20% (although we observed a smaller dropout of 10% in previous studies^25^), results in a sample size of 40 per group (total sample size = 120).

### 2.8. Data analysis

#### 2.8.1. Data reduction

Startle amplitude will be calculated by subtracting the mean baseline activity preceding the startle probe (−20 to 0 ms) from the peak muscle potential in the latency window of 31 to 150 ms. Startle responses, which are considered outliers (3 SDs from the mean), responses with eyeblink artefacts, excessive baseline activity, or other contaminations^41^ will be replaced by the average startle amplitude in the respective condition. Responses smaller than 2 × max amplitude preceding startle probe delivery (−20 to 0 ms) are classified as nonresponses and replaced with 0. Finally, the startle data will then be standardized (*z* score).^23^ Heart beat intervals, also referred to as RR intervals (intervals between peaks of QRS complex), and SCL will be reduced into 2-second bins. Changes in HR and SCL are determined by subtracting RR intervals and SCL during 2 seconds at the beginning of the anticipation phase (provocation) or before script presentation (imagery) from subsequent 2-second bins. Startle *Z* scores, changes in HR and SCL will be averaged for the anticipation, provocation, and recovery phase (provocation) and the imagery and postimagery phase (imagery) of each condition for use in inferential statistics. To determine resting high-frequency HR variability (HF-HRV) during the 5-minute resting phase at the end of the study, continuous RR-series will be dissected in 60-second bins (50% overlap), linearly detrended, and processed through an end-tapered Hamming window. The segments will then be subjected to fast Fourier transform, and HF-HRV will be calculated based on the log mean power (ms^2^) within the high-frequency band (HF-HRV; 0.15–0.40 Hz).

#### 2.8.2. Data analysis plan for the provocation paradigm

To test the hypothesis that the provocation of sensations proximal to the main pain elicits a defensive response, repeated-measures ANOVAs will be conducted separately for each physiologic measure (ie, startle magnitude, HR, and SCL) including group (CAP, CH, and HCs) as a between-subjects factor and type of provocation task (ie, tighten stomach, neck task, and neutral task) as within-subjects factor.

#### 2.8.3. Data analysis plan for the imagery paradigm

To test the hypothesis that the imagery of sensations proximal to the main pain location elicits a defensive response, repeated-measures ANOVAs will be conducted separately for each physiologic measure including group as a between-subjects factor and type of imagery script (pain-specific/fear/neutral imagery) as within-subjects factor.

## 3. Discussion

We have described 2 paradigms for investigating defence response mobilization in children with chronic pain during anticipation, exposure, and imagery of interoceptive sensations locally proximal to the main pain region. Based on models of interoceptive fear conditioning, we expect locally proximal interoceptive sensations in both paradigms (ie, experienced and imagined interoceptive sensations) to elicit a mobilization of defence responses in adolescents with chronic pain. Defence response mobilization is examined using a multimodal assessment including measures of startle modulation, changes in HR, and SCL, as well as by self-reported fear. Results based on individualized pain-specific aversive imagery scripts might inform the development of standardized individualized imagery-based exposure treatments.

Thus far, only 4 studies have investigated interoceptive exposure in children and adolescents with mixed but generally promising results.^1,20,25,49^ These studies vary, however, considerably in the form of interoceptive exposure, the symptom provocation tasks used, the implementation of imagery-based tasks, the age of the children and adolescents, the study design, the outcome variables, and apparently, the study results. Two studies implemented symptom provocation tasks^2,49^ ranging from spinning while standing, a task from the audiovestibular domain, to running down the hall with a belt fastened around the belly, a disorder-specific task. Two studies used mental imagery in the form of imagining increases in pain intensity during exposure sessions.^20,25^ We are not aware of any systematic experimental work in adolescents with CH or abdominal pain, which incorporates psychophysiological measures to examine defence response mobilization after symptom provocation or imagery-based tasks. Thus, the 2 paradigms have the potential to close this research gap. Our research will therefore have important implications for future research and clinical practice. First, interoceptive exposure treatments may be optimized by incorporating specific tasks that are able to elicit a comprehensive fear response into interoceptive exposure treatment in adolescents with chronic pain. These tasks may either constitute symptom provocation tasks or imagery-based tasks. Second, a comparison of the 2 forms of interoceptive exposure—symptom provocation and imagery-based—may be warranted to reveal which form of exposure leads to significant reductions in key variables. Third, our research might point to possible mechanisms of change during interoceptive exposure treatments, particularly regarding psychophysiological measures. Thus, it might be possible that these treatments decrease fear of pain by desensitizing defensive networks.

Some limitations of the paradigms and the study design should be considered. Observations during our ongoing study have revealed that some adolescents have difficulties to differentiate between uncomfortable innocuous stimuli (eg, pressure sensation) and pain in the imagery paradigm. From a predictive coding perspective, ambiguous or noisy sensations can be considered painful, based on top-down expectations and previous experience.^9,26^ Thus, it may be difficult for adolescents to discriminate painful from nonpainful stimuli consistently. Some studies showed that associative fear learning influences perceptual discrimination,^47,48^ and that patients with chronic pain display a deficit in discrimination of muscle tension.^21^ On the other hand, not only innocuous stimuli but also mild pain may become a CS, which predicts more intense pain (US), analogue to mild sensations (CS) predicting sensations with a higher threat value (eg, hyperventilation; US), in panic disorder.^6,24^ Furthermore, given that previous research provided some evidence for different self-reported fear responses in adolescents with CH and adolescents with CAP,^19^ our project might shed light on disorder-specific patterns in defence responses when confronted with proximal interoceptive sensations.

We have proposed 2 paradigms—*provocation and imagery*, which will shed light on the defence response of adolescents with CH and abdominal pain, when confronted with or imagining interoceptive sensations locally proximal to their main pain. Data based on our paradigms might be useful for improving existing or developing new treatments to decrease fear of bodily sensations in adolescents with CH and abdominal pain, such as interoceptive exposure or interoceptive imagery exposure.

## Disclosures

The authors have no conflict of interest to declare.
